# Ventilator associated pneumonia: comparison between quantitative and qualitative cultures of tracheal aspirates

**DOI:** 10.1186/cc2965

**Published:** 2004-10-14

**Authors:** Luis Fernando Aranha Camargo, Fernando Vinícius De Marco, Carmen Sílvia Valente Barbas, Cristiane Hoelz, Marco Aurélio Scarpinella Bueno, Milton Rodrigues Jr, Verônica Moreira Amado, Raquel Caserta, Marinês Dalla Valle Martino, Jacyr Pasternak, Elias Knobel

**Affiliations:** 1 Assistant Physican, Intensive Care Unit, Hospital Israelita Albert Einstein, São Paulo, Brasil; 2 Postgraduate Fellow, Intensive Care Unit, Hospital Israelita Albert Einstein, São Paulo, Brasil; 3 Respiratory Therapist, Intensive Care Unit, Hospital Israelita Albert Einstein, São Paulo, Brasil; 4 Microbiology Laboratory, Intensive Care Unit, Hospital Israelita Albert Einstein, São Paulo, Brasil; 5 Head, Intensive Care Unit, Hospital Israelita Albert Einstein, São Paulo, Brasil

**Keywords:** bacterial pneumonia, qualitative evaluation, quantitative evaluation, tracheal aspirates, ventilator-associated pneumonia

## Abstract

**Introduction:**

Deferred or inappropriate antibiotic treatment in ventilator-associated pneumonia (VAP) is associated with increased mortality, and clinical and radiological criteria are frequently employed to establish an early diagnosis. Culture results are used to confirm the clinical diagnosis and to adjust or sometimes withdraw antibiotic treatment. Tracheal aspirates have been shown to be useful for these purposes. Nonetheless, little is known about the usefulness of quantitative findings in tracheal secretions for diagnosing VAP.

**Methods:**

To determine the value of quantification of bacterial colonies in tracheal aspirates for diagnosing VAP, we conducted a prospective follow-up study of 106 intensive care unit patients who were under ventilatory support. In total, the findings from 219 sequential weekly evaluations for VAP were examined. Clinical and radiological parameters were recorded and evaluated by three independent experts; a diagnosis of VAP required the agreement of at least two of the three experts. At the same time, cultures of tracheal aspirates were analyzed qualitatively and quantitatively (10^5 ^colony-forming units [cfu]/ml and 10^6 ^cfu/ml)

**Results:**

Quantitative cultures of tracheal aspirates (10^5 ^cfu/ml and 10^6 ^cfu/ml) exhibited increased specificity (48% and 78%, respectively) over qualitative cultures (23%), but decreased sensitivity (26% and 65%, respectively) as compared with the qualitative findings (81%). Quantification did not improve the ability to predict a diagnosis of VAP.

**Conclusion:**

Quantitative cultures of tracheal aspirates in selected critically ill patients have decreased sensitivity when compared with qualitative results, and they should not replace the latter to confirm a clinical diagnosis of VAP or to adjust antimicrobial therapy.

## Introduction

The incidence of nosocomial pneumonia in mechanically ventilated patients ranges from 9% to 68%, and mortality rates range from 33% to 71% [1,2]. In the EPIC (European Prevalence of Infection in the Intensive Care) study [3], ventilator-associated pneumonia (VAP) was the most frequent infection acquired in the intensive care unit (ICU), accounting for 45% of all infections in European ICUs.

The diagnosis of VAP is a challenge for the clinician because the presentation is variable, and other causes of fever and chest infiltrates may occur in these patients. Clinical/radiological evaluations provide the only criteria that permit timely diagnosis. Early institution of adequate antibiotic therapy is associated with decreased mortality, at least in the more severely ill patients. Culture results are currently used to guide adjustment or withdrawal of antibiotic therapy rather than to decide whether to treat. The practice of changing therapy with culture results has resulted in reduced consumption of antibiotics. Conversely, studies have shown that over-treatment with antibiotics may select organisms such as *Pseudomonas aeruginosa *and *Acinetobacter calcoaceticus *[4,5].

The value of endotracheal aspirates for diagnosing VAP is controversial, but there is a growing body of evidence showing an important role for these cultures. Recent studies have consistently shown that outcome in VAP may not be influenced by whether cultures are obtained by bronchoscopy or from tracheal aspirates collected at the bedside. Furthermore, a cost-effectiveness analysis [6] strongly supported the employment of tracheal aspirates in the management of VAP.

Although the use of tracheal aspirates in VAP management is increasing, there are few data regarding the usefulness of quantitative as opposed to qualitative cultures. Some studies [7,8] suggested that quantitative cultures should be used in order to avoid false-positive results, but little is known about the sensitivity and specificity of quantitative culture findings in severely ill patients who have previously received broad-spectrum antibiotics.

We conducted a prospective follow up of severely ill patients in a general ICU with a high rate of antibiotic use in order to evaluate the value of quantification of bacterial colonies in tracheal aspirates for diagnosing VAP.

## Methods

### Study protocol

This study was conducted between March 2000 and January 2001 in a 28 adult bed medical/surgical critical care unit at the Hospital Israelita Albert Einstein – a major referral tertiary care centre. The ethics committee of our institution granted approval for this investigation.

During the study period, every Monday morning all patients under mechanical ventilation for at least 48 hours were examined to determine whether they had VAP by three well trained intensivists and a respiratory therapist. We chose to evaluate all ventilated patients irrespective of the presence of VAP because on Mondays we routinely perform surveillance cultures of tracheal aspirates (in a search for multidrug-resistant pathogens and to determine contact precautions for such situations). We also aimed to include both patients with and without VAP based on clinical and radiological criteria.

The diagnosis of VAP was confirmed if there was agreement between two of the three physicians using clinical/radiological criteria. On the same day, the respiratory therapist also provided a description of the appearance (purulence) of the tracheal secretions. Endotracheal secretions were collected using a standard procedure and endotracheal aspirates samples were sent for qualitative and quantitative culture. The research team was blind to culture results, but the physicians were aware of the patients' antibiotic consumption when they were evaluated.

Clinical characteristics were recorded at every evaluation (not just at enrolment in the study).

### Diagnosis of ventilator-associated pneumonia

For the purposes of the present study, VAP was diagnosed when a patient on mechanical ventilation for at least 48 hours developed a new or progressive pulmonary infiltrate on the chest radiograph in association with at least two of the following findings: râles or dullness to percussion on chest examination; new onset of purulent sputum or change in sputum character; decrease of at least 10% in arterial oxygen tension/fractional inspired oxygen ratio; leucocytes in excess of 12,000/mm^3 ^or under 4000/mm^3^; positive blood cultures or pleural effusion cultures; and axilar temperature greater than 37.8°C or under 36.0°C in the absence of antipyretic treatment (excluding another site of infection).

### Tracheobronchial aspirate samples and microbiological processing

Tracheobronchial secretions were collected by the respiratory therapist, following specimen collection guidelines, after tracheal instillation of 5 ml saline. The specimens were sent to the laboratory and cultivated within 1 hour of collection. A dilution of the tracheal aspirate was prepared and inoculated with a calibrated loop on chocolate agar and MacConkey agar. After overnight incubation in appropriate conditions, the plates were interpreted according to quantification of growth [9,10]. Qualitative cultures were considered positive when the growth of any micro-organism occurred and quantitative cultures were considered positive when the growth of 10^5 ^colony-forming units (cfu)/ml or more was observed. Sensitivity, specificity, positive predictive value and negative predictive values for qualitative and quantitative (10^5 ^cfu/ml and 10^6 ^cfu/ml) cultures from tracheal aspirates were calculated according to standard formulae. All samples were collected on the day of clinical and radiological evaluation.

## Results

A total of 106 patients were prospectively evaluated during the study period. The mean age (± standard error) was 66.6 ± 18.3 years. A total of 88 patients (83.0%) were male and 18 (17.0%) were female. The mean Acute Physiology and Chronic Health Evaluation II score was 20.1 ± 6.5. Medical patients constituted the majority (60.38%) compared with surgical patients (39.62%; Table 1). Among medical patients, 30 (28.2%) were neurological and 21 (19.8%) were cancer patients.

In these 106 patients, a total of 314 clinical evaluations were conducted and endothracheal aspirates collected, corresponding to 42.3 ± 36.5 days (mean ± standard error) of mechanical ventilation. In 95 of these evaluations the radiological or laboratory investigations for VAP were incomplete at the time of clinical evaluation, and so these evaluations were excluded. Therefore, a total of 219 evaluations in 106 patients were included in the analysis.

Thirty-eight (17.4%) evaluations were classified as 'with VAP' in 33 patients and 181 (82.6%) were classified as 'without VAP' in 73 patients (Table 2). The overall concordance between the first two observers for a diagnosis of VAP in the total population was high (94%). Within the VAP group, the overall concordance between the first two observers was 86.9%.

### Qualitative and quantitative analyses

For qualitative analysis, among all 219 evaluations, 168 (76.7%) yielded cultures that were positive for at least one agent. In the VAP group, 31 of the 38 evaluations yielded positive cultures (81.6%). Thus, the sensitivity of qualitative cultures of tracheal aspirates was 81% and the specificity was 23%. The likelihood ratio for a positive test was 1.05 and the likelihood ratio for a negative one was 0.83. The positive predictive value was 18% and the negative predictive value was 86%.

For quantitative analysis, among the 219 evaluations, 117 had = 10^5 ^cfu/ml in tracheal secretions (53.4%) and 49 had = 10^6 ^cfu/ml (22.4%). In the VAP group, 25 of the 38 evaluations had = 10^5 ^cfu/ml (65.8%) and 10 of them had = 10^6 ^cfu/ml (26.3%). Thus, for 10^5 ^cfu/ml the sensitivity was 65% and the specificity was 48%. The likelihood ratio of a positive test was 1.25 and the likelihood ratio of a negative test was 0.73. The positive predictive value was 21% and the negative predictive value was 87%. For 10^6 ^cfu/ml the sensitivity was 26% and the specificity was 78%. The likelihood ratio of a positive test was 1.18 and the likelihood ratio of a negative test was 0.95. The positive predictive value was 20% and the negative predictive value was 83% (Table 3).

In the VAP group leucocytosis was present in 26 evaluations (68.4%) and fever in 24 (63.1%), and purulent endotracheal secretions were observed by the therapist in 22 (57.8%) evaluations. In four evaluations only (10.5%) was blood culture positive for the same agent as was isolated in endotracheal secretions (Table 4).

Overall, in 96.8% of evaluations patients were receiving at least one antibiotic. Prescription of antibiotics for three or more days before data collection was high (86.7%). The most frequently administered antibiotics were glycopeptides (49.7%), antifungals (42.4%), third-generation cephalosporins (39.2%), or carbapenem (34.2%; Table 5).

Considering all VAP episodes, the most frequently isolated agents were *Staphylococcus aureus *(15.7%), *P. aeruginosa *(15.7%) and *Acinetobacter baumanii *(7.3%). Fungi accounted for 13.3% of all agents isolated. In 18.4% of evaluations in the VAP group, no agent was recovered from the endotracheal aspirates (Table 6).

### Clinical observations

Considering the population as a whole, in 59 evaluations (26.9%) patients had a tracheostomy. Stress ulcer prophylaxis was present at 210 of the 219 evaluations (96%), with H_2_-receptor blockers in 58.4%, proton pump inhibitors in 36.5% and sucralfate in 0.9%. Sepsis was diagnosed in 46 (21%) evaluations.

Among the 38 evaluations classified as positive for VAP, tracheostomy was present in ten (26.3%). Previous lung disease was observed in six (15.7%) events. Ulcer prophylaxis was present in 100% of evaluations, with H_2_-receptor blockers in 22 (57.8%) and proton pump inhibitors in 16 (42.2%). Sepsis was diagnosed in 14 (36.8%) evaluations.

Other clinical characteristics are listed in Table 2. A total of 31 (29.2%) patients died during their hospitalization: 11 (33.3%) of the 33 patients in the VAP group and 20 (27.3%) of the 73 patients without VAP (not significant).

## Discussion

VAP is the most frequent type of infection in ICU patients in Europe and Latin America (almost half of all nosocomial infections) [3] and ranks second in US ICUs [11]. The attributable mortality is higher in medical than in surgical patients, and rates vary according to the case mix and aetiological agent [12].

Inadequate or delayed antimicrobial treatment in VAP is an established independent predictor of death [13]. According to published data, changing an initial empirical treatment based on subsequent culture results may have either a beneficial effect (in terms of mortality, less antibiotic use, less days on antibiotics) [14] or no effect in more severely ill patients [15]. For this reason, efforts must be directed at choosing adequate empirical treatment as early as possible, which may be accomplished with a high degree of suspicion and adequate guidelines based on local antibacterial susceptibilities. In addition, adhering to ideal pharmacological principles (choosing continuous as opposed to intermittent administration, adjustment for renal and hepatic failures), reducing dosages when appropriate, and shortening the duration of treatment are presently standard of care for VAP.

In order to avoid any delay in instituting antibiotic treatment, reliable diagnostic methods should be employed. Despite their variable sensitivity and specificity [16], clinical/radiological findings may currently be considered the best option, although rapid tests, such as the percentage of infected leucocytes on bronchial specimens, are promising in that they can provide rapid confirmation [17]. Culture results for bronchial or tracheal samples may be available late in the course of an episode of VAP and should not be used to decide whether to treat, especially in patients who are severely ill. On the other hand, culture results should be used to adjust (narrow or extend antibiotic spectrum) or withdraw empirical treatment – a practice that has been shown to be beneficial, with no increase in mortality, and that directs medical staff to seek other unsuspected foci of infection [18].

Although bronchoscopic samples increase the degree of confidence that a diagnosis of VAP is correct [14], endotracheal aspirates, despite their lack of consistency as a diagnostic tool [19], are widely employed in the management of VAP. Recent small trials have consistently shown that there is no advantage of using bronchoscopic methods over relying on tracheal aspirate cultures when mortality is an end-point [6,20,21]. Reduced costs and similar outcomes were reported using either quantitative or qualitative tracheal aspirates for guiding or deciding to interrupt antibiotic treatment for VAP [6]. This may be due to the high correlation between tracheal aspirates (both quantitative and qualitative) and bronchoscopic cultures when presence of VAP is highly probable [21,22]. However, the above-mentioned studies did not determine the value of quantification of micro-organisms in tracheal aspirate samples as compared with qualitative assessment.

Quantification of micro-organisms in biological samples for the purpose of diagnosing infectious conditions is widely used, particularly for nosocomial infections. Regarding respiratory infections, bronchoscopic samples have established cutoff values (10^4 ^cfu/ml for bronchoalveolar lavage [BAL] fluid and 10^3 ^cfu/ml for protected brush specimen [PBS]) for improving diagnostic performance. On the other hand, use of these cutoff values has yielded conflicting results, and previous antibiotic treatment has great impact on these values. Souweine and coworkers [23] showed that the standard cutoff values of BAL and PSB would have to be lowered to 10^3 ^cfu/ml and 10^2 ^cfu/ml to retain diagnostic accuracy where antibiotics were previously administered, mainly when they are given in the preceding 24 hours.

Only a small number of studies have evaluated the role of quantitative endotracheal cultures in the diagnosis of VAP. Albert and coworkers [24], studying 20 ventilated patients and using clinical/radiological parameters, found the threshold of 10^5 ^cfu/ml to have a sensitivity of 81%, specificity of 65%, positive predictive value of 55% and negative predictive value of 55%. In that study different cutoff values were not tested to evaluate the real usefulness of quantification. Jourdain and coworkers [25] studied a group of 57 patients with presumed VAP, 19 (33%) of whom were confirmed by PSB sample with more than 10^3 ^cfu/ml. Using quantification in this population, those investigators showed that the sensitivity of the test reduced considerably from 86% to 43% whereas specificity increased from 52% to 95% when a cutoff of 10^3 ^cfu/ml was compared with one of 10^7 ^cfu/ml. No data regarding previous use of antibiotics were available to explain the decreased sensitivity.

We conducted a prospective follow up of severely ill patients with a high rate of antimicrobial use prior to diagnosis of VAP. Not surprisingly, the most frequent agents recovered were multidrug-resistant agents, such as methicillin-resistant *S. aureus*, *P. aeruginosa *and *Acinetobacter *spp.

We found different levels of sensitivity (81%, 65%, 26%) and specificity (23%, 48%, 78%) for qualitative and quantitative (cutoffs 10^5 ^cfu/ml and 10^6 ^cfu/ml) findings, respectively, as was expected. However, the positive (18%, 21%, 20%) and negative (86%, 87%, 83%) predictive values obtained were very similar.

Our data reveal sensitivity values for tracheal aspirates similar to those observed in the above-mentioned studies, although specificity values were lower. According to our data, use of the cutoff value 10^5 ^cfu/ml reduced the sensitivity of the test to levels too low to be useful in clinical practice, bearing in mind the proposed role of tracheal aspirates to guide antibiotic withdrawal or modification. Moreover, quantification did not improve predictive values for the purposes of diagnosing VAP at the time when a suspected case was evaluated.

Patient characteristics may have an impact on the accuracy of diagnostic tests. Although there is broad correlation between the number of bacterial colonies in biological samples and the occurrence of infection as opposed to colonization, the exact bacterial count cannot be predicted in highly ill patients, for whom a lower inoculum may be sufficient for disease development. This has been observed for catheter-related infections in severely ill patients in a surgical ICU [26], in which true catheter-related bacteraemia was reported with fewer than 15 cfu on catheter tips. In our patient population there was a significant proportion of patients with renal failure, diabetes, cancer and sepsis – conditions that are known to be associated with immunosuppression.

These decreased sensitivity values may also be explained by antimicrobial use. More than 95% of the patients studied were receiving antibiotics when the sample was collected for analysis, and the majority of them were broad-spectrum antibiotics (almost 50% had received glycopeptides and 35% carbapenems). About 80% had received them for longer than 72 hours. Decreased accuracy of quantification with samples obtained by bronchoscopy was reported by Soweine and coworkers [23]. BAL and PSB had significantly less sensitivity when the procedure was performed within 24 hours of antibiotic use than when antibiotics had not been given for longer than 72 hours. The impact of antibiotic use may be greater for tracheal aspirates, irrespective of the timing of administration; this may be due to the higher concentration of the antibiotic in upper tract secretions, although this point requires further investigation.

Our study has a number of limitations. While we attempted to achieve a high degree of certainty in clinical/radiological parameters, with the participation of three experienced ICU physicians (with a high degree of correlation between them), no 'gold standard' technique was employed, such as bronchoscopic samples (although it remains controversial whether bronchoscopy samples can be regarded as the gold standard for VAP). Because of the low specificity of clinical judgement, we must consider the fact that we are studying a population in which VAP rate is over-estimated. This is supported by the rate of 18.4% of VAP diagnoses with a negative tracheal aspirate finding and a 13.3% rate of fungal isolates, which only rarely can be considered true causative agents. Thus, it is possible that we have false-positive rate of at least 31.7%, although technical problems with specimen collection cannot be ruled out. The virtual absence of a gold standard for VAP makes study designs that address the issue of diagnostic tests difficult. In accordance with our study design, we evaluated all patients with mechanical ventilation every week, irrespective of clinical suspicion of VAP. This strategy may have beneficial effects because we included in the same population patients who were likely and those who were unlikely to have definite VAP, but increasing the possibility of false-positive cases.

Other study designs use populations selected because clinical/radiological judgement suggest the presence of VAP. In these studies, the control cases (no VAP) are defined as having negative bronchoscopic cultures, based on predetermined cutoff values. In these situations, problems with the lesser sensitivity of bronchoscopic samples in patients on antibiotics, and even the intrinsically low sensitivity of this diagnostic strategy when compared with histological criteria [27], increase the likelihood of including false-negative control individuals. In other words, with our study design we might have overestimated VAP, as compared with underestimating it with conventional study designs. For this reason we think that there is no ideal design for such studies, and studies that rely solely upon clinical/radiological parameters should not systematically be discarded. Furthermore, the use of bronchoscopy in our hospital is unreliable, as it may be in a large number of general ICUs.

Tracheal aspirates have a definite role to play in the management of VAP, but only when correlated with clinical findings [28]. The use of quantitative results may be associated with under-diagnosis of VAP, leading to inappropriate changes to antibiotic regimens and, in some cases, antibiotic delay or withdrawal.

## Conclusion

The severely ill and those who have previously received courses of broad-spectrum antibiotics – a population whose number is expected to increase in modern ICUs – may be targeted for use of qualitative findings rather than quantitative cultures of tracheal secretions for VAP management. Quantitative results may add costs and workload (in our laboratory it is five times more time consuming) and may then be of limited value in this group of patients, although enhanced specificity may be beneficial in terms of avoiding unnecessary treatment. In selected groups of severely ill patients, quantitative cultures of tracheal aspirates should not replace qualitative cultures for confirmation of diagnosis or management of antibiotic therapy.

## Key messages

• 	Quantitative cultures of tracheal aspirates have increased specificity compared with qualitative analysis for diagnosis of VAP.

• 	The sensitivity values for quantitative cultures of tracheal aspirates are significantly lower than those for qualitative cultures for VAP diagnosis in severely ill patients receiving prior antibiotics.

• 	Quantitative cultures of tracheal aspirates should not replace qualitative cultures for the purpose of confirming a clinical diagnosis of VAP or adjusting antimicrobial therapy.

## Competing interests

The authors declare that they have no competing interests.

## Abbreviations

BAL = bronchoalveolar lavage; cfu = colony-forming unit; ICU = intensive care unit; PSB = protected specimen brush; VAP = ventilator-associated pneumonia.

## Figures and Tables

**Table 1:** Demographic data of the patients investigated

Parameter	Value
Number of patients	106
Age (years)	66.6 ± 18.3
Ratio of males to females (*n*)	88/18
APACHE II score	20.1 ± 6.5
Clinical category (*n *[%])	
Medical	64 (60.3%)
Cancer	21 (19.8%)
Neurological	30 (28.2%)
Surgical	42 (39.7%)

APACHE, Acute Physiology and Chronic Health Evaluation.

**Table 2:** Clinical characteristics of the patients in the events investigated.

Parameter	Total evaluations (%)	Evaluations in VAP group (%)
Number of evaluations	219 (100%)	38 (100%)
Tracheostomy (*n *[%])	59 (26.9%)	10 (26.3%)
Atelectasis^a ^(*n *[%])	13 (6.0%)	6 (15.7%)
Lung edema^a ^(*n *[%])	35 (16.0%)	9 (23.6%)
Lung contusion^a ^(*n *[%])	6 (2.7%)	1 (2.6%)
Pleural effusion^a ^(*n *[%])	21 (9,5%)	8 (21.0%)
Previous lung disease (*n *[%])	30 (13.6%)	6 (15.7%)
COPD	16 (7.3%)	3 (7.8%)
Cancer	11 (5.0%)	2 (5.2%)
Asthma	2 (0.9%)	1 (2.6%)
Pulmonary fibrosis	1 (0.4%)	None
Broncoaspiration (*n *[%])	12 (5.4%)	3 (7.8%)
Sepsis (*n *[%])	46 (21.0%)	14 (36.8%)
ARDS (*n *[%])	12 (5,4%)	3 (7.8%)
Renal failure (*n *[%]; creatinine >2.0 mg/dl)	91 (41.5%)	20 (52.6%)
Diabetes (*n *[%])	35 (16.0%)	5 (13.1%)
Chemotherapy (*n *[%])	13 (6.0%)	2 (5.2%)
Radiotherapy (*n *[%])	2 (0.9%)	None
Immunossupressants drugs (*n *[%])	5 (2.2%)	1 (2.6%)
AIDS (*n *[%])	3 (1.3%)	None
Renal transplantation (*n *[%])	5 (2.2%)	1 (2.6%)
Abdominal surgery (*n *[%])	32 (14.6%)	7 (18.4%)
Multiple trauma (*n *[%])	21 (9.5%)	3 (7.9%)
Neuromuscular blocking agents (*n *[%])	7 (3.1%)	7 (18.4%)
Central venous line (*n *[%])	215 (98.0%)	38 (100%)
Intracranial pressure monitoring (*n *[%])	20 (9.1%)	1 (2.6%)

^a^According to clinical judgement. ARDS, acute respiratory distress syndrome; COPD, chronic obstructive pulmonary disease; VAP, ventilator-associated pneumonia.

**Table 3:** Qualitative and quantitative analysis

Parameter	Qualitative	Quantitative	
		
		10^5 ^cfu/ml	10^6 ^cfu/ml
Sensitivity	81%	65%	26%
Specificity	23%	48%	78%
Positive predictive value	18%	21%	20%
Negative predictive value	86%	87%	83%
Likelihood ratio of positive test	1.05	1.25	1.18
Likelihood ratio of negative test	0.83	0.73	0.95

cfu, colony-forming units.

**Table 4:** Diagnostic criteria for ventilator-associated pneumonia in order of occurrence

Diagnostic criteria	*n *(%)
Leukocytosis	26 (68.4%)
Fever	24 (63.1%)
Purulent tracheal secretion	22 (57.8%)
Decrease of at least 10% in PaO_2_/FiO_2 _ratio	16 (42.1%)
Rales or dullness to percussion on chest examination	9 (23.6%)
Leucopenia	4 (10.5%)
Blood positive cultures	4 (10.5%)
Hypothermia	2 (5.2%)

FiO_2_, fractional inspired oxygen; PaO_2_, arterial oxygen tension.

**Table 5:** Prescription of antimicrobials in all the events studied

Class of antimicrobial	Evaluations (*n *[%])
Glycopeptide	109 (49.7%)
Antifungical	93 (42.4%)
Third generation cephalosporin	86 (39.2%)
Carbapenem	75 (34.2%)
Clindamicin	34 (15.5%)
Quinolone	31 (14.1%)
Metronidazol	26 (11.8%)
Fourth generation cephalosporin	23 (10.5%)
Macrolides	23 (10.5%)
Total	212 (96.8%)

**Table 6:** Infectious agents isolated in the evaluations of patients with ventilator-associated pneumonia

Aetiological agents	Evaluations (*n *[%])
Gram-negative bacteria	15 (39.4%)
Gram-positive bacteria	11 (28.9%)
Negative cultures	7 (18.4%)
One or more agents	6 (15.7%)
Fungus	5 (13.3%)
Isolated agents	
*Staphylococcus aureus*	6 (15.7%)
*Pseudomonas aeruginosa*	6 (15.7%)
*Acinetobacter baumanii*	3 (7.8%)
